# Transforming Experience: The Potential of Augmented Reality and Virtual Reality for Enhancing Personal and Clinical Change

**DOI:** 10.3389/fpsyt.2016.00164

**Published:** 2016-09-30

**Authors:** Giuseppe Riva, Rosa M. Baños, Cristina Botella, Fabrizia Mantovani, Andrea Gaggioli

**Affiliations:** ^1^Applied Technology for Neuro-Psychology Laboratory, Istituto Auxologico Italiano, Milan, Italy; ^2^Centro Studi e Ricerche di Psicologia della Comunicazione, Università Cattolica del Sacro Cuore, Milano, Italy; ^3^Universidad de Valencia, Valencia, Spain; ^4^Instituto Salud Carlos III, Ciber Fisiopatología Obesidad y Nutrición (CB06/03), Madrid, Spain; ^5^Red de Excelencia PROMOSAM, Mineco, Spain; ^6^Universitat Jaume I, Castelló de la Plana, Spain; ^7^Università degli Studi Milano Bicocca, Dipartimento di Scienze Umane per la Formazione “Riccardo Massa”, Milan, Italy

**Keywords:** virtual reality, augmented reality, personal change, anxiety disorders, eating disorders, acute pain, post-traumatic stress disorder, body swapping

## Abstract

During life, many personal changes occur. These include changing house, school, work, and even friends and partners. However, the daily experience shows clearly that, in some situations, subjects are unable to change even if they want to. The recent advances in psychology and neuroscience are now providing a better view of personal change, the change affecting our assumptive world: (a) the focus of personal change is reducing the distance between self and reality (conflict); (b) this reduction is achieved through (1) an intense focus on the particular experience creating the conflict or (2) an internal or external reorganization of this experience; (c) personal change requires a progression through a series of different stages that however happen in discontinuous and non-linear ways; and (d) clinical psychology is often used to facilitate personal change when subjects are unable to move forward. Starting from these premises, the aim of this paper is to review the potential of virtuality for enhancing the processes of personal and clinical change. First, the paper focuses on the two leading virtual technologies – augmented reality (AR) and virtual reality (VR) – exploring their current uses in behavioral health and the outcomes of the 28 available systematic reviews and meta-analyses. Then the paper discusses the added value provided by VR and AR in transforming our external experience by focusing on the high level of personal efficacy and self-reflectiveness generated by their sense of presence and emotional engagement. Finally, it outlines the potential future use of virtuality for transforming our inner experience by structuring, altering, and/or replacing our bodily self-consciousness. The final outcome may be a new generation of transformative experiences that provide knowledge that is epistemically inaccessible to the individual until he or she has that experience, while at the same time transforming the individual’s worldview.

## Introduction: Understanding Personal Change

How can we use technologies like virtual reality or augmented reality to support personal and clinical change? A meaningful answer to this question requires an in-depth examination of the process of change. During our life, we undergo many personal changes: we change our house, our school, our work, and even our friends and partners. However, in this paper, we will focus on a peculiar type of change – personal change – whose main effect is a change in the conceptual system of the subject (assumptive world), derived from perceptions of one’s own behavior or experience, or other incoming information (1). Personal change plays an adaptive role in managing symptoms of distress produced by life transitions and traumatic events (1, 2). Moreover, high levels of personal change are associated with psychological well-being (2).

Our daily experience shows clearly that, in some situations, subjects are unable to change even if they want to. To help these subjects, clinical psychology is often used to facilitate personal change. However, as noted by Higginson and Mansell (3): “The mechanism of change is not fully understood. This is clear in research demonstrating the efficacy of different therapeutic approaches and the significant rates of natural recovery” (p. 326). On one side different studies suggest the lack of differential effectiveness between therapies – many therapies have equivalently positive outcomes – despite manifestly non-equivalent theories and techniques (4, 5). On the other side, some people experience personal change without the help of any form of psychotherapeutic treatment.

The recent advances in psychology and neuroscience are now providing a better view of personal change that help us in understanding the potential of these technologies. The main tenets are:

1. *change is contextual*: depending on the person, the issues, and the situation (6);
2. *the self can be both a barrier and a catalyst to change*: people are motivated to maintain self-integrity (7);
3. *change is a process*: it happens in discontinuous and non-linear ways, following life transitions and traumatic events (8).

We will deepen these points in the following section.

The starting point for our exploration of the process of change is the perceptual control theory (PCT) (9, 10). According to this vision, the process of control is the critical feature of human nature (9): “life is a constant process of comparing how things are with how we want things to be, and if they do not match doing something to get closer to how we want things to be” (p. 250).

Generally, control is a defined as the process of reducing the distance between what we want and what we are (*error*). Interestingly, the source of errors is both *within* and *between* individuals (9, 11). Specifically, PCT suggests that a possible source of error is internal: the coherence between goals and subgoals of the individual (*conflict*).

To eliminate a conflict, the individual must direct his or her awareness to the experience that is creating the conflict. Then, a reorganization is required: a trial and error process, which modifies the characteristics or the conflicting goals (3).

The PCT can be integrated with the vision of a second theory: self-affirmation theory (SAT) (12, 13). According to this view, individuals are motivated to maintain their self-integrity, defined as (7) “a sense of global efficacy, an image of oneself as able to control important adaptive and moral outcomes in one’s life” (p. 336). On one side, any threat to it evokes self-defense and psychological stress. On the other side, subjects can import into a critical domain the sense of personal integrity that they feel in another. In the concept of self-integrity, a critical role is played by self-efficacy, the strength of one’s belief in one’s own ability to complete tasks and reach goals (14, 15). As noted by Bandura (16, 17), self-efficacy determines whether the process of change will be initiated, how much effort will be expended toward it, and how long it will be sustained in the face of obstacles and aversive experiences.

The process of change is also the focus of another vision: the TransTheoretical Model of Behavior Change (18–20). This model describes personal change as a progression through a series of five stages: precontemplation, contemplation, determination, action, and maintenance. These stages represent a temporal dimension that allows both the individuals and the persons supporting them to understand when particular shifts in attitudes, intentions, and behaviors occur.

However, not all personal changes occur in a linear or gradual manner. As noted by Miller and C’de Baca (21), individuals may have “transformative experiences” able to produce a deep and enduring restructuration of one or more personal dimension. According to Mezirow’s Transformative Learning Theory (22, 23), these experiences can be triggered by a “disorienting dilemma” usually related to a life crisis or major life transition (e.g., death, illness, separation, or divorce), which forces individuals to critically examine and eventually revise their core assumptions and beliefs. The outcome of a transformative experience is a significant and permanent change in the expectations – mindsets, perspectives and habits of mind – through which we filter and make sense of the world.

As noted by Kottler (6) and Riva (24), by merging these theories, we can identify some important properties of personal change:

- the focus of personal change is reducing the distance between self and reality (conflict);
- this reduction is achieved through: (a) an intense focus on the particular experience creating the conflict and (b) an internal or external reorganization of this experience;
- this process can be the outcome of either a sudden transformative experience or a progression through a series of different stages:
  - selfhood is affected by a crisis, trauma, or developmental transition;
  - a level of pain and discomfort is reached that cannot any longer be ignored or denied;
  - there is an awareness or insight that something different must be done (change);
  - there is a process of applying what was realized or learned into new meanings and/or constructive action;
  - if a sufficient level of action is achieved, it alters the perception of the environment and sets new goals; and
  - there is recovery from inevitable relapses.

The critical steps in this process are three: the emergence of transformative experiences, the passage between stage *c* and *d* and the one between stage *d* and *e*.

As noted by Gaggioli (25), transformative experiences provide knowledge that is epistemically inaccessible to the individuals until they have that experience. For this reason, transformative experiences cannot be planned in advance but happen suddenly in individuals’ lives without a prior control on their contents and their effects.

Instead, the passage between stage *c* and *d* requires *self-reflectiveness*: an intense focus on the particular instance or experience creating the conflict (26). By exploring this experience as thoroughly as possible, the individual can relive and identify all of the significant elements associated with it (e.g., conceptual, behavioral, emotional, and motivational) facilitating their reorganization (24).

Finally, the passage between stage *d* and *e* requires the belief of *personal efficacy* (16, 17): individuals have to believe that they have the power to effect changes through their actions. Without it there, they are not willing to act, or to keep on acting in the face of problems and difficulties.

Starting from these premises, the paper wants to review the potential of virtuality for enhancing the process of personal change. First, the paper will explore the two leading virtual technologies – augmented reality (AR) and virtual reality (VR) – assessing their current uses in behavioral health and the outcomes of the available systematic reviews and meta-analyses. Then the paper will discuss their added value in transforming our external experience by focusing on the elevated level of personal efficacy and self-reflectiveness generated by their sense of presence and emotional engagement. Finally, it will outline the potential future use of virtuality for transforming our inner experience by structuring, altering, and/or replacing our bodily self-consciousness (BSC).

## The Virtual Technologies: Augmented Reality and Virtual Reality

### Experiential Learning through Augmented Reality

**Figure 1:**
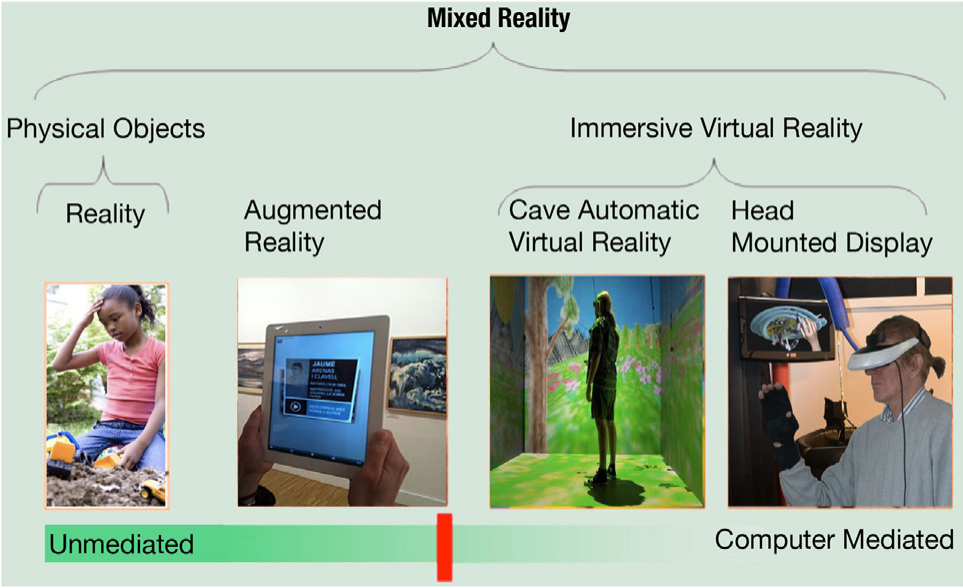
**The reality–virtuality continuum [adapted from Milgram and Kishino (30)]**.

Augmented reality can be described as an interactive visualization system (a head-mounted display, a computer, a game console, a smartphone, or a tablet) allowing the merging of digital contents with the real environment surrounding the user (27, 28). In simpler words, AR allows the augmentation of our real experience blending both “real-world elements” and “virtual elements,” which may involve not only the view but also hearing, touch, and smell (29). For this reason, within the reality–virtuality continuum (see Figure 1) introduced by Milgram and Kishino (30) to describe all the possible combinations of real and virtual objects, AR is the step just after the real environment.

In fact, the most important feature of AR is that the synthetic objects and data provide the real world with remarkable and valuable information for its user. van Krevelen and Poelman describe this opportunity in the following way (31): “Imagine a technology with which you could see more than others see, hear more than others hear, and perhaps even touch, smell, and taste things that others cannot. What if we had technology to perceive completely computational elements and objects within our real world experience… that help us in our daily activities, while interacting almost unconsciously through mere gestures and speech?” (p. 1).

The additional information offered by AR can be a powerful tool for personal change, because it can support and improve the sense of self-reflectiveness and personal efficacy of its users.

For these features, AR seems to be a promising and useful tool for intervention in the treatment of specific phobias (29, 32), as also supported by a recent systematic review (28) (see Table 1) and a narrative review (33).

**Table 1:** **Meta-analyses and systematic reviews related to the use of AR in the different areas of behavioral health**.

	Review type	Reference	Included studies	Conclusions (from papers)
Anxiety disorders	Systematic review	(28)	13 studies	“In general, the presented studies show that the AR seems to be a promising and useful tool for intervention in the treatment of specific phobias. Nevertheless, the small sample of subjects examined, and the lack of control group and randomized controlled studies necessitate more randomized controlled experiments for exploring the AR efficacy in the clinical treatments”

**Figure 2:**
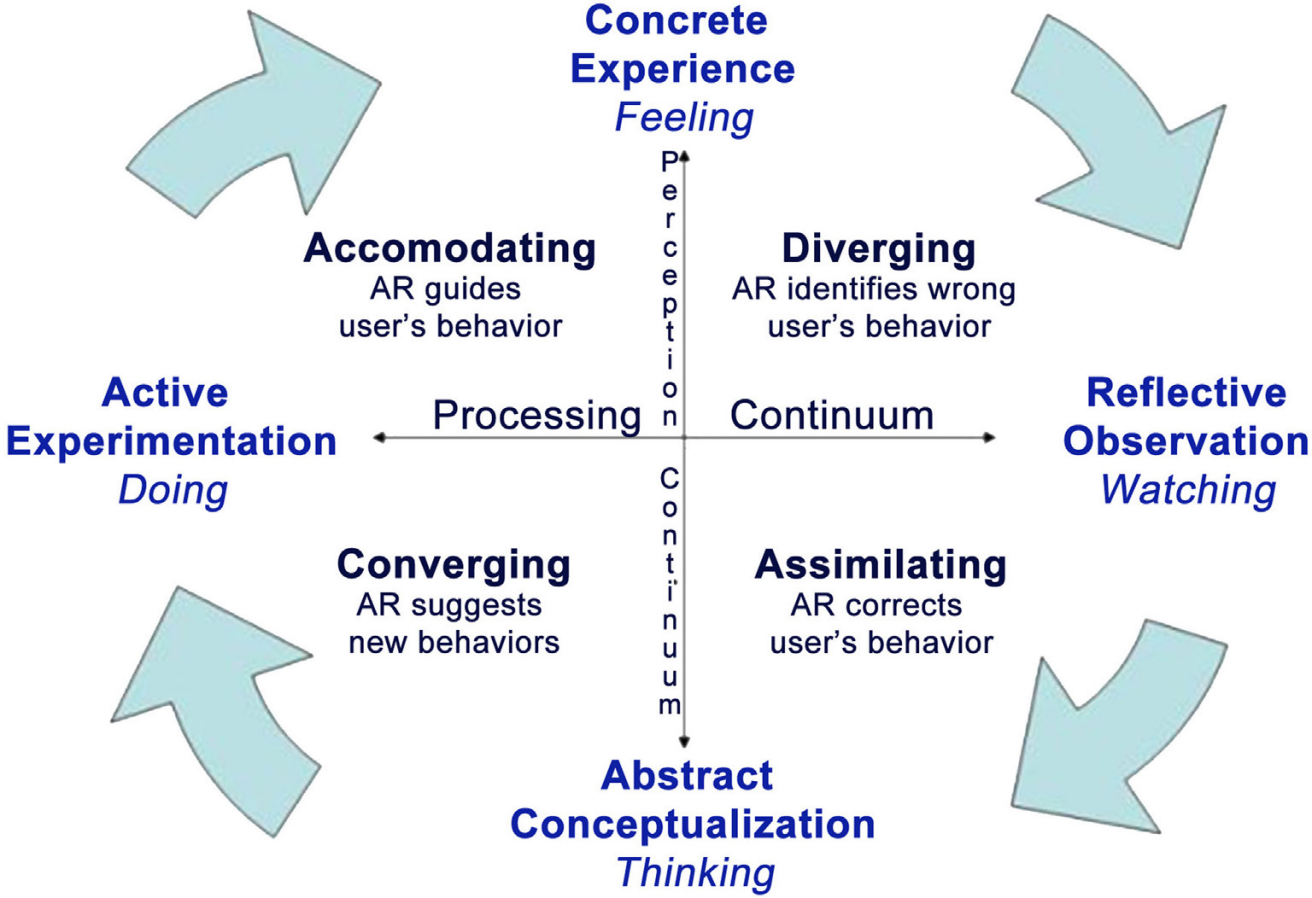
**The role of AR in the experiential cycle [adapted from Kolb (40)]**.

However, its potential in supporting personal change is wider as demonstrated by different emerging applications: from post-stroke (34) and physical rehabilitation (35, 36), social (37) and emotional (38) training for children with autism, to pain reduction (39). Here, we suggest that the added value of AR is related to the support it offers to all the stages of the experiential learning cycle (40) (see Figure 2).

According to the influential model introduced by David Kolb, experiential learning is a process consisting of four stages: experience, observation and reflection, abstract reconceptualization, and experimentation (40, 41). The individual starts the learning process from his/her experience, which leads to observations and reflections on its contents. Specifically, abstract conceptualization is used to create a generalization of the experience that is evaluated, integrated with the available knowledge, and converted into recommendations. These recommendations activate new actions and strategies, which can be tested and explored, to adjust the original experience.

A possible example of how AR can be used to support experiential learning in relation to clinical change is the treatment of a specific phobia, for example cockroach phobia (32, 42, 43). In the “concrete experience” stage, the patient observes the cockroaches in the “here-and-now” offered by AR. This experience is the “basis for observation and reflection” (reflective observation): the patient has the opportunity to consider the actions – e.g., avoidance – and emotions – e.g., irritability and fear – experienced and to think about his/her behavior in previous similar experiences (generalization). At this stage, the role of the therapist is important: generalization questions – e.g., individual is asked to compare the performance in earlier exposures and to identify the pros and cons of the different behaviors – can be used to facilitate abstract conceptualization and to identify recommendations supporting the next exposure. During the new exposure, AR can support, in real time, the patient, offering real-time information about his/her status – e.g., the level of emotional arousal – and practical suggestions – e.g., to keep their hands closer to the ones of the therapist. Moreover, AR can enhance the process of change throughout the entire treatment, for instance, Botella and colleagues (44) used an AR-based serious game in a mobile phone in order to facilitate the exposure treatment. The goal of this game was reducing the level of fear and avoidance before the AR exposure session and promoting the over-learning after the AR exposure session as a homework assignment.

In this way, AR facilitates personal change through a cyclical interaction of experience, thought, and reflection (active experimentation).

In other words, AR is the perfect experiential learning tool. On one side, it allows real-time interactivity in an ecological setting improving concentration and motivation (45). On the other side, it provides targeted and non-directive suggestions and guidelines that help users to develop skills and knowledge in a more effective way (46). Finally, as suggested by Baus and Bouchard (32), AR can be used in the actual places the subject encounters his difficulties, facilitating the transfer of the acquired skills to the real world.

### Virtual Reality as Simulative Technology

Within the reality–virtuality continuum (see Figure 1) introduced by Milgram and Kishino (30), VR is the final step, in opposition to reality: if AR adds digital information to the real-world environment, VR completely replaces it with a virtual one. But what is VR?

In computer sciences, VR is usually described as a set of fancy technologies (47, 48): an interactive 3D visualization system (a computer, a game console, or a smartphone) supported by one or more position trackers and head-mounted display. The trackers sense the movements of the individual and report the collected data to the visualization system, which updates the scene in real time.

However, in psychology and neuroscience, VR is instead defined as (49) “an advanced form of human–computer interface that allows the user to interact with and become immersed in a computer-generated environment in a naturalistic fashion” (p. 82). From a psychological perspective, VR is a *subjective experience* cheating the individual out of the illusion that he/she is there, that this experience is real (50). Specifically, VR is different from other media because it induces the sense of “presence”: the feeling of “being there” inside the virtual experience produced by the technology (51, 52).

While still is lacking a general consensus about the definition and the etiology of presence [for an introduction to this topic see Ref. (53–66)], most researchers agree about what it is not (65). As underlined by Riva and colleagues (54) “Presence is not the degree of technological immersion, it is not the same thing as emotional engagement, it is not absorption or attention or action; but all of these have a potential role in understanding the experience of presence in interaction – the experience of interacting with presence” (p. 1).

The sense of presence offered by VR can be a powerful tool for personal change because it offers *a world where the individual can stay and live a specific experience* (47, 67, 68). Following the model of personal change discussed before, VR allows a level of *self-reflectiveness* that is higher than the one provided by memory and imagination, and it is more controlled than the one offered by direct “real” experience. In fact, VR can also be defined as an “*advanced imaginal system*” (69–71): an experiential form of imagery that is as effective as reality in inducing emotional responses.

As underlined by Glantz and colleagues (72) “One reason it is so difficult to get people to update their assumptions is that change often requires a prior step – recognizing the distinction between an assumption and a perception. Until revealed to be fallacious, assumptions constitute the world; they seem like perceptions, and as long as they do, they are resistant to change” (p. 96). Using the sense of presence induced by VR, it is easier to develop new, realistic, credible, and informative experiences regarding the surrounding world or the self demonstrating to the individual that what is assumed to be true – e.g., my team disapproves me – in fact is a result of his/her mind. Once this has been understood, it is easier to identify all of the elements supporting the assumption and make them available for reorganization (73).

These features clearly explain the increasing use of VR in behavioral health and, in particular, in the treatment of anxiety disorders. In Table 2, we reported all the available systematic reviews and meta-analyses related to the use of VR in behavioral health. To select them, a computer-based search in several databases was performed for relevant publications. Databases used for the search were PsycINFO, PubMed/Medline, and Web of Science (Web of Knowledge). We searched using the string “Virtual Reality” AND (“review” OR “systematic review” OR “meta-analysis” OR “meta-analysis”). English, French, and German were used as language limits. More, we hand-searched the reference lists of all relevant articles to find additional studies (snowball technique).

**Table 2:** **Meta-analyses and systematic reviews related to the use of VR in the different areas of behavioral health**.

	Review type	Reference	Included studies	Conclusions (from papers)
**Addictions**	Systematic review	Bordnick et al. (90)	14 studies	“Research using VR has shown that drug-dependent people react with strong craving to specific cues (e.g., cigarette packs and liquor bottles) as well as environments or settings (e.g., bar and party) associated with drug use. Virtual reality has also been used to enhance learning and generalization of relapse prevention skills in smokers by reinforcing these skills in lifelike environments”
	Systematic review	Hone-Blanchet et al. (91)	21 studies	“VR enhances ecological validity of traditional craving-induction measurement. Specifically, findings indicate that VR can successfully increase craving. Studies combining cue-exposure therapy with virtual environment, however, reported mitigated success so far”
	Meta-analysis	Pericot-Valverde et al. (94)	18 studies	“Presentations of smoking cues through virtual reality can produce strong increases in craving among cigarette smokers. This strong cue-reactivity effect, which was comparable in magnitude to the craving effect sizes, found with more conventional modes of cue presentation, supports the use of virtual reality for the generation of robust cue-specific craving in cue-reactivity research”

**Anxiety disorders**	Meta-analysis	Parsons and Rizzo (74)	21 studies	“Although meta-analysis revealed large declines in anxiety symptoms following VRET, moderator analyses were limited due to inconsistent reporting in the VRET literature”
	Meta-analysis	Powers and Emmelkamp (75)	13 studies	“Analysis showed a large mean effect size for VRET compared to control conditions, Cohen’s *d* = 1.11 (SE = 0.15, 95% CI: 0.82–1.39). This finding was consistent across secondary outcome categories as well (domain-specific, general subjective distress, cognition, behavior, and psychophysiology). Also, as expected, *in vivo* treatment was not significantly more effective than VRET. In fact, there was a small effect size favoring VRET over *in vivo* conditions, Cohen’s *d* = 0.35 (SE = 0.15, 95% CI: 0.05–0.65)”
	Systematic review	Meyerbroker and Emmelkamp (76)	20 studies	“Only in fear of flying and acrophobia, there is considerable evidence that VRET indeed is effective. In more complex anxiety disorders as panic disorder and social phobia, which form the core clinical groups, first results of VRET are promising, but more and better controlled studies are needed before the status of empirically supported treatment is reached. More severe cases of panic disorder with agoraphobia and social phobia are often not reached with existing treatments”
	Meta-analysis	Opris et al. (77)	23 studies	“The results show that VR does far better than the waitlist control, and similar efficacy between the behavioral and the cognitive behavioral interventions incorporating a VR exposure component and the classical evidence-based interventions. VR has a powerful real-life impact, similar to that of the classical evidence-based treatments, and a good stability of results over time, similar to that of the classical evidence-based treatments. There is a dose–response relationship for VRET, and there is no difference in the dropout rate between the VR exposure and the *in vivo* exposure”
	Systematic review	McCann et al. (78)	27 studies	“VRET may be an effective method of treatment but caution should be exercised in interpreting the existing body of literature supporting VRET relative to existing standards of care. The need for well-designed VRET research is discussed”
	Meta-analysis	Ling et al. (66)	33 studies	“Analysis showed a medium effect size for the correlation between sense of presence and anxiety (*r* = 0.28; 95% CI: 0.18–0.38). Moderation analyses revealed that the effect size of the correlation differed across different anxiety disorders, with a large effect size for fear of animals (*r* = 0.50; 95% CI: 0.30–0.66) and a no to small effect size for social anxiety disorder (*r* = 0.001; 95% CI: −0.19 to 0.19). Further, the correlation between anxiety and presence was stronger in studies with participants who met criteria for an anxiety disorder than in studies with a non-clinical population”
	Systematic review	Diemer et al. (62)	38 studies	“Despite several limitations, this review provides evidence that VR exposure elicits psychophysiological fear reactions in patients and healthy subjects, rendering VR a promising treatment for anxiety disorders, and a potent research tool for future investigations of psychophysiological processes and their significance during exposure treatment”

**Stress-related disorders**	Systematic review	Goncalves et al. (80)	10 studies	“The results suggest the potential efficacy of VRET in the treatment of PTSD for different types of trauma. VRET proved to be as efficacious as exposure therapy. VRET can be particularly useful in the treatment of PTSD that is resistant to traditional exposure because it allows for greater engagement by the patient and, consequently, greater activation of the traumatic memory, which is necessary for the extinction of the conditioned fear”
	Systematic review	Serino et al. (81)	10 studies	“VR-based cyber-SIT cyber-SIT may play an important role in the future clinical psychology, but it is crucial to enhance the validation of this approach from a methodological point of view: controlled trials testing a greater number of participants are needed”
	Systematic review	Motraghi et al. (82)	9 studies	“Although preliminary findings suggest some positive results for VRET as a form of exposure treatment for PTSD, additional research using well-specified randomization procedures, assessor blinding, and monitoring of treatment adherence is warranted. Movement toward greater standardization of treatment manuals, virtual environments, and equipment would further facilitate interpretation and consolidation of this literature”
	Systematic review	Botella et al. (83)	12 studies	“Results suggest VR is effective in the treatment of PTSD. Not all studies reported having followed the clinical guidelines for evidence-based interventions in the treatment of PTSD. Few studies evaluated acceptability, however, the findings are very promising, and patients reported high satisfaction and acceptability regarding the inclusion of VR in the treatment of PTSD. The main weaknesses identified focus on the need for more controlled studies, the need to standardize treatment protocols using VR, and the need to include assessments of acceptability and related variables”

**Autism**	Systematic review	Aresti-Bartolome and Garcia-Zapirain (96)	11 studies	“Virtual reality makes it possible to create safe environments where they can learn rules and repeat the tasks. Furthermore, interacting with avatars where social situations are replicated enables patients to work on these situations and find more flexible solutions. This means that virtual environments may be good instruments to work on social skills with ASD sufferers”
	Systematic review	den Brok and Sterkenburg (97)	28 studies	“Specific kinds of technologies can be used to learn specific kinds of skills (e.g., videos on computers or handheld devices for daily living skills; virtual reality for time perception and emotions of others). For attaining cognitive concepts, advanced technologies such as virtual reality are effective”
**Depression**	Systematic review and meta-analysis	Li et al. (105)	19 studies	“The unique experience of virtual reality exposure therapy was reported to be particularly effective for reducing depression caused by fear. The meta-analysis revealed a moderate effect size of the game interventions for depression therapy at posttreatment [*d* = −0.47 (95% CI −0.69 to −0.24)]”

**Eating disorders and obesity**	Systematic review	Ferrer-Garcia and Gutierrez-Maldonado (114)	12 studies	“Although examined results suggest that VR-based therapy is an effective intervention for treating body image disturbances, more controlled studies with larger clinical samples are needed”
	Systematic review	Koskina et al. (116)	4 studies	“Data indicate that using virtual environments provide alternative ways of delivering exposure therapy that has promising outcomes. Overall, it is possible that VR may be a useful intervention for ED, and its implementation is recommended either as a stand alone treatment or as an intermediary step prior to *in vivo* exposure”
	Systematic review	Ferrer-Garcia et al. (115)	17 studies	“Although several methodological deficiencies were detected in the reviewed studies, there is fair evidence for the effectiveness of VR-based treatments in ED and obesity. VR-based interventions usually combine exposure to VR environments with cognitive therapies. The VR component seems to be especially suitable for reducing body image disturbances and for increasing self-esteem and self-efficacy”

**Pain reduction**	Systematic review	Morris et al. (109)	9 studies	“VR, in conjunction with pharmacologic analgesics, significantly reduced pain experienced by burn injury patients during wound dressing changes and physiotherapy. There is equivocal evidence for the effect of VR in conjunction with pharmacologic analgesics on reducing anxiety in burn injury patients during wound dressing changes and physiotherapy”
	Systematic review	Malloy and Milling (110)	11 studies	“VR distraction was shown to be effective for reducing experimental pain, as well as the discomfort associated with burn injury care. Studies of needle-related pain provided less consistent findings”
	Systematic review	Triberti et al. (112)	11 studies	“Results suggest the importance of different psychological factors in the effectiveness of the analgesic distraction. While sense of presence influences the effectiveness of VR as a distraction tool, anxiety as well as positive emotions directly affect the experience of pain”
	Systematic review	Garrett et al. (111)	17 studies	“There was strong overall evidence for immediate and short-term pain reduction after VR, whereas moderate evidence was found for short-term effects on physical function. Little evidence exists for longer-term benefits”

**Psychosis**	Systematic review	Valmaggia et al. (106)	16 studies	“The review identified studies investigating the effect of interpersonal sensitivity, childhood bullying victimization, physical assault, perceived ethnic discrimination, social defeat, population density, and ethnic density on the real-time appraisal of VR social situations. Further studies demonstrated the potential of VR to investigate paranoid ideation, anomalous experiences, self-confidence, self-comparison, physiological activation, and behavioral response. The reviewed studies suggest that VR can be used to investigate psychological processes and mechanisms associated with psychosis”

**Schizophrenia**	Meta-analysis	Välimäki et al. (107)	3 studies	“There is no clear good quality evidence for or against using virtual reality for treatment compliance among people with schizophrenia. If virtual reality is used, the experimental nature of the intervention should be clearly explained. High-quality studies should be undertaken in this area to explore any effects of this novel intervention and variations of approach”
	Systematic review	Veling et al. (108)	4 studies	“There is a small but expanding literature on interventions for delusions, hallucinations, neurocognition, social cognition, and social skills; preliminary results are promising. VR applications for assessment and treatment of psychotic disorders are in their infancy but appear to have a great potential for increasing our understanding of psychosis and expanding the therapeutic toolbox”

We have included only articles on VR used for supporting personal and clinical change. Excluded from the analysis were studies related to the use of VR in surgery, physical and cognitive rehabilitation, and review articles lacking basic information about the selection of the discussed papers.

Our initial search yielded 918 non-duplicate citations screened *via* PsycINFO, PubMed/Medline, and Web of Science (Web of Knowledge). After the application of inclusion/exclusion criteria, papers have been reduced to 67 articles. A more in-depth investigation of the full papers resulted in an exclusion of 40 articles. Twenty-seven of them were excluded because their specific focus was not VR, while the remaining 13 were excluded, because they lacked a clear description of the process used to select the discussed papers. In the end, 27 studies met full criteria and were included in Table 2.

As expected, the highest number of papers – four meta-analyses and seven systematic reviews – is related to the use of VR in the treatment of emotion-related disorders: anxiety disorders (four meta-analyses and three systematic reviews) (66, 74–79) and stress-related disorders (four systematic reviews) (80–83). The results support the use of VR in the treatment of phobias (66, 74, 75, 79), stress management (81), post-traumatic stress disorders (80, 82, 83), and panic disorders with or without agoraphobia (77). No definitive evidence is available for the treatment of social phobia (66, 75, 76). More, as underlined by most studies (84), and specifically by McCann and colleagues (78), the quality of future research has to be improved using well-specified randomization procedures, assessing treatment adherence, and providing a better standardization of clinical protocols.

As noted by Riva and Mantovani (85), the rationale behind the use VR in anxiety disorders is simple “…in VR, the patient is intentionally confronted with the feared stimuli while allowing the anxiety to attenuate. Avoiding a dreaded situation, reinforces a phobia, and each successive exposure to it reduces the anxiety” (p. 21). In other words, VR is a versatile tool that permits to develop multiple environments that can be presented to the user in many different forms (66, 86). Recent studies show that VR exposure to multiple contexts reduces the recurrence of fear to a greater extent than exposure to only one scenario (87); in the same way, return of fear at posttreatment was significantly reduced by the use of multiple stimuli contexts during exposure (33, 88). Therefore, exposure to different virtual contexts can be an effective way to generalize the results. More, as suggested by Diemer and colleagues (62), VR can be used to induce emotional reactions *via* different routes (perceptual vs. conceptual), with additive effects if combined.

These studies are in agreement with the results obtained by Craske et al. (89) on the inhibitory learning approach presenting exposure optimization strategies such as (a) “deepened extinction,” where multiple fear stimuli are first extinguished separately before being combined during extinction; (b) “variability,” using different stimuli, levels of intensity, and durations; or (c) “exposure to multiple contexts,” different in terms of colors and textures. These kinds of effects are not easy to obtain in the “real world” but easier to achieve by using VR.

A similar process can be used in addiction. As underlined by two systematic review by Bordnick and colleagues (90) and Hone-Blanchet and colleagues (91), VR stimuli (e.g., liquor bottles and cigarette packs) and VR environments (e.g., bar and party) are effective in inducing strong craving in cocaine/alcohol/smoking-dependent subjects. It is supposed that the reactions induced by VR cues support motivational processes reducing relapse in addicts attempting to remain abstinent (92, 93). The same result is reported in the recent meta-analysis by Pericot-Valverde and colleagues exploring the use of VR in cigarette craving assessment. The paper confirms the potential of VR “for the generation of robust cue-specific craving in cue-reactivity research” (94). As discussed before, the use of VR exposure allows patients to experience arousal and reactivity in a controlled setting, and to develop new coping skills through repeated exposures and practice (95).

The use of VR as a controlled setting in which to develop new skills through trials and errors is also effective with persons with autistic spectrum disorder. The two systematic reviews support the use of VR to learn how to cope with social situations (96, 97), in particular to learn communication and imitation skills.

In summary, as underlined by Pla-Sanjuanelo and colleagues (98) “the capability of developing a large amount of realistic controlled stimuli and, simultaneously, of monitoring the responses generated by the user offers a considerable advantage over real experiences” (p. 145). For example, if an individual experiences a significant fear when exposed to heights, using a virtual elevator simulation, the therapist can assure him/her that this threat will be experienced only when he/she is prepared to cope with it. The same can be said for all the elements that are present in the situation, which can make it more or less threatening (99, 100). For instance, the height of the spaces, the presence of protecting elements, and the duration of a determined situation.

More, VR allows the construction of “virtual adventures” in which subjects experience themselves as competent and efficacious (47, 101, 102). Specifically, it is possible to design targeted VR experiences with different difficulty levels – from easy performances to very difficult ones – that offer an important source of personal efficacy. Interacting with them individuals discover that the conflicts and/or feared situations can be overcome through confrontation and effort (85).

Finally, as recently suggested by Gaggioli (25), a further opportunity offered by VR is the possibility of simulating impossible worlds – that is, worlds that do not conform to the fundamental laws of logic and nature. For example, Friedman and colleagues used VR to give people the illusion of backwards time travel allowing them to relive a sequence of events in which they can intervene and change history. In this view, time alterations and time paradoxes (e.g., the possibility of changing and restructuring the history) represent a kind of impossible manipulation of physical reality that is feasible in virtual reality and that can be used to elicitate transformative experiences (103).

### Virtual Reality as Embodied and Transformative Technology

However, VR is more than a tool to provide exposure, training, and desensitization (104), as evidenced by the other areas of behavioral health in which VR systematic reviews have been found: *depression, psychosis, schizophrenia, pain management, obesity, and eating disorders*.

The first three applicative areas are still in their infancy. The systematic review discussing the use of VR in depression (105) and psychosis (106) suggests that VRET may be effective both for reducing depression caused by fear and for investigating psychological processes and mechanisms associated with psychosis. However, actual studies are not conclusive (105, 106). A similar picture is provided by the two systematic reviews assessing the use of VR in schizophrenia (107, 108): there is no clear good quality evidence for or against using VR for treatment compliance among people with schizophrenia (107), even if existing studies suggest a potential for increasing our understanding of psychosis (108). The situation is different for pain management and the treatment of obesity and eating disorders.

The four systematic reviews related to the use of VR in pain management support its use in the treatment of acute pain (109–112) and, in particular, in reducing the pain experienced by burn injury patients during wound dressing changes (109, 110). A strong overall evidence has been found for immediate and short-term pain reduction after VR, while moderate one for short-term effects on physical function. A recent systematic review tried to shed some light on the rationale of this approach (112). The results suggest that if on one side, the sense of presence influences the effectiveness of VR as a distraction tool, on the other side anxiety as well as positive emotions directly affect the experience of pain. The potential of VR in the treatment of pain was recently confirmed by “The Italian Consensus Conference on Pain in Neurorehabilitation” (113). In their analysis of the psychological interventions and psychotherapies that can be used within an integrated approach for patients undergoing neurological rehabilitation for pain, authors included the use of VR (grade of recommendation: D) for acute pain management (113).

A similar outcome is provided by the three systematic reviews related to the use of VR in the treatment of obesity and eating disorders (114–116): both VR cue exposure to food stimuli (115–117) and VR body image treatments (114, 115) are effective. A recent narrative review (117) confirms these data concluding that “VR has, for the past two decades, proven to be a useful adjunctive tool for both assessment and treatment of patients with eating disorders and obesity” (p. 71). In particular, as underlined by Gutiérrez-Maldonado and colleagues (118): “Recent studies indicate that … VR can integrate and extend existing treatments for eating and weight disorders. Future possibilities for VR to improve actual approaches include its use for altering in real time the experience of the body (embodiment) and as a cue exposure tool for reducing food craving” (p. 148).

But what is the link between the use of VR in pain and eating/weight disorders? Both are effective in modifyng the experience of the body of their users. Let us try to deepen this claim.

Virtual reality can be defined as an “embodied technology” for “its ability of modifying the feeling of presence” (119–121): in VR, subjects can experience the synthetic environment as if it was “their surrounding world” (*incarnation*: the physical body is within a virtual environment) or can experience their synthetic avatars as if they were “their own body” (*embodiment*: the physical body is replaced by the virtual one). In other words, the VR user is present in a virtual world or in a virtual body through the alteration of the cognitive factors regulating our experience of body and space [for an in-depth analysis of this claim, see Ref. (119)]. The side effect of this alteration is the experience of simulation sickness experienced by some VR users: the conflict between the visually perceived movement in the virtual world and the vestibular system’s sense of movement (I stand still) may produce negative effect like vomiting, discomfort, disorientation, and fatigue (122).

The increasing interest of cognitive science and social psychology for the experience of the body – Bodily Self-Consciousness – BSC – is providing a better picture of these processes.

First, BSC is apparently experienced by the subject as a single and coherent experience. However, neuroimaging and clinical data suggest that BSC is the outcome of different experiential layers (123–127). Specifically, we become aware of our bodies through exteroceptive signals arising outside the body (e.g., vision, and touch) and through proprioceptive (e.g., skeletal joints/muscles) and interoceptive (e.g., heart rate) signals arising inside the body (128, 129).

Second, the above studies support also the idea that body representations play a central role in structuring cognition and the self (124, 130–132). As underlined by Blanke (124) in his paper for Nature Reviews Neuroscience: “Human adults experience a “real me” that “resides” in “my” body and is the subject (or “I”) of experience and thought. This aspect of self-consciousness, namely the feeling that conscious experiences are bound to the self and are experiences of a unitary entity (“I”), is often considered to be one of the most astonishing features of the human mind” (p. 556). For this reason, the experience of the body is strictly connected to processes like cognitive development and autobiographical memory.

Third, we use the “feelings” from the body to sense both our physical condition and emotional state. These feelings range from bodily changes both visible (e.g., posture, touch, and facial expressions) and invisible (e.g., heart rate, endocrine release, and muscle contractions) to an external observer (133).

Fourth, the characteristics of BSC evolve over time following the ontogenetic development of the subject. As suggested by Riva (134), we expand over time our BSC by progressively including new experiences – minimal selfhood, self-location, agency, body ownership, third-person perspective, and body satisfaction – based on different and more sophisticated bodily representations that progressively integrate.

Fifth, bodily representations are usually produced and modulated by sensory inputs, but they can exist and produce qualitatively rich bodily experiences even in the absence of any input signal (e.g., phantom limb syndrome) (135). In this view, the experience of our bodily self is the outcome of a multimodal simulation. As underlined by Wilson (136): “The human perceptual system incorporates an emulator… that is isomorphic to the human body…the emulator draws on body-schematic knowledge derived from the observer’s representation of his own body” (p. 221).

Starting from these premises in 2007, two European teams of cognitive neuroscientists independently reported in Science how VR could be used for altering BSC (producing an out-of-body experience) in healthy volunteers (137, 138). Since then, the rapid development of immersive VR environments has allowed a new research line – *virtual embodiment* – (139–142) whose results are discussed in two recent reviews (143, 144). But how the experience of “being” in a synthetic body is achieved in these studies? These experiments are an evolution of the trick used in the low-tech rubber-hand illusion (145): the cross-modal congruence between what a person feels *via* the somatosensory pathways (touch) and what (s)he sees in VR. Using VR, different authors induced the illusion of a fake hand (146) or a fake limb (147) and produced an out-of-body experience (137) by modifying the normal association between touch and vision. Slater and colleagues even used VR to induce a body transfer illusion (147): they transferred a group of male subjects in a life-sized virtual human female body. Interestingly, altering the experience of the body has significant effects also on social cognition: for example, the transfer illusion in a body of different race produced a significant reduction of the implicit bias against that race (148).

But, how can these research data drive a new generation of VR tools aimed at supporting personal and clinical change? Up to now, VR has been used to simulate external reality, which is to make people feel “real” what is actually not really there. The next step is the use of VR for the simulation of our internal reality, including the way we perceive our body, control it, and affectively react to what happens to it. The final outcome is a new generation of transformative experiences that provide knowledge that is epistemically inaccessible to the individual until he or she has that experience, while at the same time transforming the individual’s worldview (25).

This opportunity may also open a radically new research field in medicine – Embodied Medicine – allowing new clinical solutions for the treatment of neurological and psychiatric disorders where our BSC seems to be altered (119). Considerable evidence suggests that the etiology of different disorders – including PTSD, eating disorders, depression, chronic pain, phantom limb pain, autism, schizophrenia, Parkinson’s and Alzheimer’s – may be related to an impaired/altered BSC. More, it may offer a scientific path to improve the level of well-being in non-clinical subjects by inducing positive emotions, improving attitudes, and helping individuals in understanding and controlling the signals of their body.

In general, it is possible to modify our BSC in three different ways (see Table 1) (25, 119, 120, 149, 150):

• *By structuring BSC through the focus and reorganization of its contents (mindful embodiment)*: individuals have different levels of body awareness is the extent of sensitivity and attentiveness to bodily signals and sensations (151). VR, if integrated with other technologies like biosensors, can be applied for improving body awareness. For example, in integration with biofeedback, training can be used to assess and control specific body signals like heart rate, galvanic skin response, electromyography, or electroencephalography (152, 153).
• *By augmenting BSC to achieve enhanced and extended experiences by altering/extending its boundaries (augmented embodiment)*: by integrating VR with biosensors, stimulation, and haptic devices, it is possible to map the contents of a sensory channel to a different one – e.g., vision to touch or to hearing – for augmenting its sensibility and replacing the impaired channels (150).
• *By replacing the contents of BSC with synthetic ones (synthetic embodiment)*: as we have seen before, VR allows different type of synthetic bodily experiences. The most advanced is the “*full body swapping*” in which the individual’s body is substituted by a virtual body (154). In other words, as in the movie *Being John Malkovich*, using VR, individuals can experience the perspective of another individual by seeing what the other see, hearing what the other hear, and touching what the other touch (155, 156). In a recent attempt of applying this method, Serino and colleagues (157) successfully used the illusion of body ownership over a body different to current one (a virtual body with a skinny belly) with a non-operable extreme obese patient (e.g., body mass index >60 kg/m^2^). Their data show that, after body swapping, the patient reduced the levels of body dissatisfaction and body distortion. More, she increased her motivation for undertaking healthy behavior and decreased the level of anxiety feelings associated with her clinical condition.

## Conclusion

This paper claimed that virtual technologies – AR and VR – have the potential for supporting personal and clinical change: if AR adds virtual information to the real world, VR completely replaces the real environment with a virtual one. Specifically, both AR and VR can transform our external experience through the high level of personal efficacy and self-reflectiveness generated by their sense of presence and emotional engagement. Moreover, VR can also modify our inner experience by structuring, altering, and/or replacing our BSC.

In the first part of the paper, we explored the characteristics of the process of change. By integrating the available literature, we identified three critical steps that may slow down or block the process.

First, the process of change requires *self-reflectiveness*: an intense focus on the particular instance or experience creating the conflict (26). By focusing on this experience as much as possible, the individual can relive and identify any significant element (e.g., conceptual, behavioral, emotional, and motivational) facilitating its reorganization (24).

Second, the process of change requires *personal efficacy* (16, 17): individuals have to believe that they have the power to effect changes through their actions. Without it there, they are not willing to act or to keep on acting in the face of problems and difficulties.

Finally, the process of change can be dramatically boosted by *transformative experiences* (22, 23), forcing individuals to critically examine and eventually revise their core assumptions and beliefs. The key advantage of these experiences is that they are epochal (158): “a sudden, dramatic, and reorienting insight” (p. 86) pushing the individual to an immediate and irreversible change. Unfortunately, most transformative experiences cannot be planned in advance, but happen suddenly in individuals’ lives, without a prior control on their contents and their effects.

In the second part of the paper, we analyzed the outcomes of the available systematic reviews and meta-analyses related to the use of virtual technologies in behavioral health to identify and discuss the added values offered by them.

The only available systematic review for AR support its use in the treatment of phobias. Moreover, it also outlines the value of AR as experiential learning tool. On one side, it offers real-time interactivity in an ecological setting improving concentration and motivation (45). On the other side, it provides targeted and non-directive suggestions and guidelines helping individuals to develop skills and knowledge in a more effective way (46). Finally, AR can be used in the actual places where the subject encounter his difficulties, facilitating the transfer of the acquired skills to the real world (32).

The 27 meta-analyses and systematic reviews available for VR support the use of this technology in the treatment of anxiety disorders, stress-related disorders, obesity and eating disorders, and pain management. But still, there is no clear good quality evidence for or against using VR for the treatment of depression and schizophrenia.

In most pathologies, VR is used as simulative tool for controlled exposure to critical/fearful situations. The possibility of presenting realistic controlled stimuli and, simultaneously, of monitoring the responses generated by the user offers a considerable advantage over real experiences. More, the possibility of designing targeted VR experiences with different difficulty levels – from easy performances to very difficult ones – offers an important source of personal efficacy.

However, the use of VR in pain management and in the treatment of obesity and eating disorders suggest a different rationale: VR can also be used as an embodied technology able to alter our experience of the body and space. If most VR applications to date have been used to simulate external reality, it is also possible to use VR for the simulation of our internal reality including the perception and ownership of our body. The final outcome may be a new generation of transformative experiences that provide knowledge that is epistemically inaccessible to the individual until he or she has that experience, while at the same time transforming the individual’s worldview and pushing him/her to an immediate and irreversible personal or clinical change (25, 159). More, it may offer a scientific path to improve the level of well-being in non-clinical subjects by inducing positive emotions, improving attitudes, and helping individuals in understanding and controlling the signals of their body.

At the end, the contents of this review offer a sound foundation and rationale for researchers interested in using virtual technologies for improving personal and clinical change.

## Author Contributions

GR performed the literature review and drafted the first version of the manuscript. RB, CB, FM, and AG supervised the rationale and the scientific contributions. All authors read and approved the final manuscript.

## Conflict of Interest Statement

The authors declare that the research was conducted in the absence of any commercial or financial relationships that could be construed as a potential conflict of interest.
